# GDIS, a global dataset of geocoded disaster locations

**DOI:** 10.1038/s41597-021-00846-6

**Published:** 2021-02-16

**Authors:** Elisabeth L. Rosvold, Halvard Buhaug

**Affiliations:** 1 Department of Economic History and International Relations, Stockholm University, Stockholm, Sweden; 2 Peace Research Institute Oslo, PRIO, Oslo, Norway; 3 Department of Sociology and Political Science, Norwegian University of Science and Technology, NTNU, Trondheim, Norway

**Keywords:** Environmental social sciences, Natural hazards, Geography, Social sciences

## Abstract

This article presents a new open source extension to the Emergency Events Database (EM-DAT) that allows researchers, for the first time, to explore and make use of subnational, geocoded data on major disasters triggered by natural hazards. The Geocoded Disasters (GDIS) dataset provides spatial geometry in the form of GIS polygons and centroid latitude and longitude coordinates for each administrative entity listed as a disaster location in the EM-DAT database. In total, GDIS contains spatial information on 39,953 locations for 9,924 unique disasters occurring worldwide between 1960 and 2018. The dataset facilitates connecting the EM-DAT database to other geographic data sources on the subnational level to enable rigorous empirical analyses of disaster determinants and impacts.

## Abstract (summary)

**Table:** 

Measurement(s)	geographic location • geocoded disaster locations
Technology Type(s)	digital curation • geocoding
Factor Type(s)	year that the disaster has occurred
Sample Characteristic - Environment	climate system
Sample Characteristic - Location	global • subnational administrative areas

Machine-accessible metadata file describing the reported data: 10.6084/m9.figshare.13177022

## Background & Summary

Existing global databases of the prevalence and intensity of disasters provide important policy-relevant insights into major trends across space and time^1^ and permit aggregate studies of how disasters relate to, inter alia, anthropogenic climate change^2^ and quality of governance^3^, as well as their macroeconomic consequences^4^. However, the aggregate nature of these databases, which typically lack precise subnational information on disaster locations, prevents systematic empirical investigations of, e.g., how, why, where, and when natural hazards transform into disasters across contexts, and how drivers of local vulnerability determine the consequences of hazard exposure. Until now, researchers have been able to respond to such questions only for selected cases or at an aggregate level. A more complete, global understanding of these processes requires better data at local scales^5^.

This article presents a new open source extension to the Emergency Events Database (EM-DAT)^6^ that allows researchers, for the first time, to explore and make use of subnational, geocoded data on major disasters triggered by natural hazards since 1960. EM-DAT, maintained by the Centre for Research on the Epidemiology of Disasters (CRED) at the Catholic University of Louvain in Belgium, constitutes a comprehensive and widely used multi-disaster catalogue. A search for the term “EM-DAT” in Google Scholar returns more than 17,000 hits, testimony to its widespread application in academic work. The EM-DAT database records disaster events by country and “location” (i.e., a string variable providing the names of affected provinces, districts, towns, etc.), but the database contains no geographical information that allows easy integration into a geospatial analysis framework.

The absence of quantified location data (geometries) complicates detection and analysis of spatiotemporal patterns of disaster impacts, and further hinders connecting the information available in the EM-DAT database with georeferenced third-party data, such as localized economic^7^, demographic^8^, and security conditions^9^ in order to understand the relation between disasters and affected societies’ underlying vulnerability^10^. Disaster hazards, vulnerabilities, and exposure are all inherently local^11,12^, and a range of subnational investigations exist for single countries and specific disasters^13^. The dataset presented in this article adds to the increasing pool of spatially and temporally high-resolution data sources.

The Geocoded Disasters (GDIS) dataset provides spatial geometries in the form of GIS polygons as well as centroid latitude and longitude coordinates for each administrative entity listed as a disaster location in the EM-DAT database. In total, the dataset contains spatial information on 39,953 locations for 9,924 disasters occurring worldwide between 1960 and 2018. Whereas the official EM-DAT statistics provide crucial insights into general disaster patterns and trends across space and time for policy and the public, this new extension is targeted towards scientific users who need more precise information on disaster events for, e.g., impact assessments and analysis of coping capacity determinants. The utility of geocoded disasters for geographical validation of exposure has been noted for some time already^14^, and the data presented here allows such analyses also beyond the insurance sector and permits users to conduct spatially sensitive impact assessments.

While consequence-based disaster measures are in themselves endogenous to the vulnerability of affected societies^15^, this extension can be used to explore, and to some extent remedy, this concern. A promising avenue in this regard is comparing the new geolocated disasters with high-resolution hazard exposure data to better understand the conditions under which extremes are especially likely to translate into material and human disasters. Another promising future avenue is combining the new disaster data with household surveys to explore variation in micro-level impacts of disasters and whether the implementation of adaptation programs or provision of development aid increases local resilience to weather extremes^16,17^.

## Methods

In EM-DAT, all disasters are coded at the country level with one row, or unique observation, per disaster event and affected country. Information on subnational location is provided in the form of a string ‘Location’ variable that lists the name(s) of affected administrative area(s) of each event (usually at the first- or second-order administrative level, such as district, province or state). The database also contains ‘Latitude’ and ‘Longitude’ columns that provide coordinates for the named location in some instances. However, for the large majority of disaster events, these columns are left blank, and even in cases with multiple named affected administrative areas, at most only one pair of coordinates is provided. To remedy this shortcoming, we ascribe spatial information to all relevant disasters by means of polygon (outline) shapes of affected first- and (whenever possible) second- and third-order subnational administrative units, stored in a geographic information systems (GIS) format. In addition, each disaster location is assigned geographical coordinates denoting the centroid of the respective polygons. Of course, disasters are unlikely to affect the entire area of an administrative region in a similar fashion, so the polygons necessarily are crude approximations of the true impact zones. However, the geocoding precision is bounded by the level of detail provided in the underlying EM-DAT database, and this limitation notwithstanding, GDIS provides superior spatial information on the location of disasters worldwide over the past half century.

The GDIS dataset includes the dominant geophysical, meteorological, hydrological, and climatological disaster types: floods, storms, earthquakes, volcanic activity, extreme temperatures, landslides, droughts, and (dry) mass movements. Due to declining quality of information in earlier periods, we decided to limit the geocoding to disaster events occurring since 1960.

In order to facilitate the geocoding, we first needed to reshape the data structure such that each named location for each disaster event becomes a separate observation, interlinked via the EM-DAT database’s event identifier (*disasterno*). The geocoding is based on the names of locations listed in the “Location” variable in EM-DAT. This is a string containing the known locations of the disaster, and can vary from being empty (missing) to containing several locations at different scales (village, region, river, historical area, etc.) with varying degrees of geographic precision.

Converting the 11,081 selected disasters into unique locations resulted in a candidate list of more than 47,000 observations. Next, the list of locations was sent through an automated script that matched the EM-DAT location names with those provided in the Global Administrative Areas (GADM) database v.3.6^18^ to obtain geometry of the affected areas. Around half of the observations got a perfect match through the automated query. Unmatched locations were then inspected manually in order to identify and reconcile cases with spelling inconsistencies between EM-DAT and GADM. This procedure reduced the number of unmatched disaster event locations further. Remaining observations (typically names of specific locations or geographical features, such as villages or rivers, that do not feature in the GADM database) were cross-checked against Google Maps and, wherever possible, ascribed to the corresponding first-, second, and third-order administrative unit. This was done both to maximize internal consistency among the geocoded disaster events and to give preference to more certain locations at higher levels of aggregation over unclear or fuzzy local places. In cases where the location was not found, the location referred to a poorly defined geographical area (for example a river flowing through large parts of a country) or the name of the location referred to multiple distinct locations within the same country, the observation was dropped. In case of inconsistencies between Google Maps and GADM, we ascribe to the latter, meaning that also the list returned from the automated query was cross-checked to match GADM boundaries.

For some disasters, the subnational location information in EM-DAT is at a more aggregate level than first-order administrative units, typically referring to a quadrant of the affected country. In such cases, the disaster events were split up into unique observations for each first-order administrative unit covered by the aggregate description. In just under 10% of the cases, the named localities lack official recognition and clear boundaries (e.g., the Hindu Kush mountain range in Central Asia), the names are vague or unknown or refer to multiple places in the same country, or the locations lack spatial geometry in the GADM database (notably for small island states), all of which prevent allocation of more accurate geometry and disaster coordinates. In the event that the location variable lists several locations at different levels of aggregation, only the most disaggregated observation was kept, as this information also provides info on all available administrative units.

Over the past half century, several countries have been dissolved (e.g., Yugoslavia, Soviet Union), others have emerged (e.g., Bangladesh, Eritrea, South Sudan), and the configuration and spatial delineation of subnational political entities have changed in most countries. Since digital time-varying boundary data of subnational administrative units are unavailable for most countries, the geocoding instead refers to the regions of the contemporary world (i.e., those represented in GADM v.3.6). A similar approach is adopted by many other providers of subnational data, including the UN Population Division, whose historical estimates and future projections are based on a snapshot of the current delimitation of political boundaries.

**Fig. 1:**
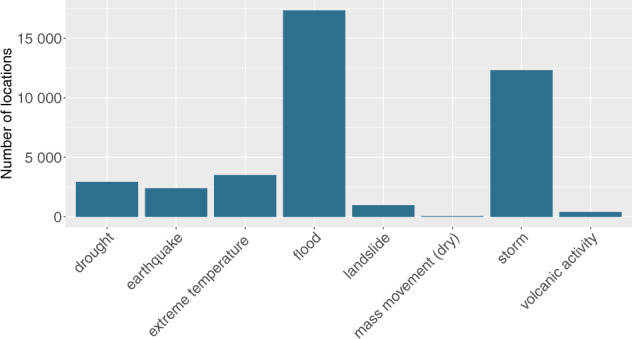
Geocoded disaster locations by type, 1960–2018. This graph was created by combining spatial information in GDIS with matching data on disaster type from EM-DAT.

In all, the geolocation project has coded spatial information on 39,953 unique disaster locations in the form of administrative unit polygons and centroid coordinates for 9,924 relevant disasters occurring between 1960 and 2018. Figure 1 shows the distribution of observations across the disaster types included in GDIS. In total, we were able to determine at least one location for 89.5% of the disasters we obtained from EM-DAT. In terms of type, droughts are at the lower end with 64% of the events being geocoded, while storms mirror the average at 89.5%. Floods and earthquakes are at the top of the list with shares of 92% and 97% respectively, indicating a skew towards rapid-onset disasters.

**Fig. 2:**
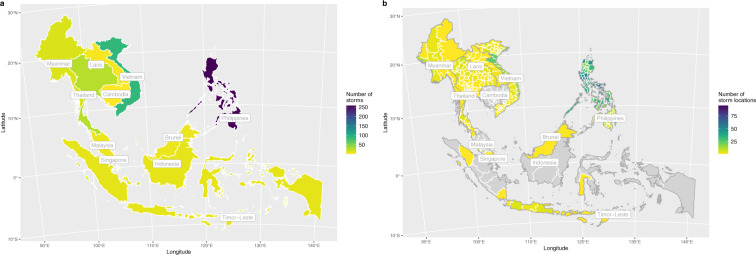
Storm-related disaster exposure across South-East Asia, 1960–2018. Grey areas denote provinces without recorded storm-related disasters in the period. Panel a shows country-aggregated statistics, whereas Panel b presents the same statistics for sub-national provinces.

To illustrate the utility of having more precise geographic information about the locatlity of disasters, Fig. 2 shows storm exposure across South-East Asia across (a) and within (b) countries. The maps clearly illustrate that the subnational exposure to storms vary significantly in most countries, notably Vietnam and the Philippines.

## Data Records

Upon registration in Earthdata, the data is publicly available through the data web portal of the Socioeconomic Data and Applications Center (SEDAC) at Columbia University^19^. SEDAC is one of the Distributed Active Archive Centers (DAACs) in the Earth Observing System Data and Information System (EOSDIS) of the U.S. National Aeronautics and Space Administration (NASA) and serves as an information gateway between earth sciences and social sciences. The dataset is stored in four different formats; geopackage and geodatabase for use with GIS software, R-file for open-source use and.csv format for ease of use in any statistical software. The latter does not include any geometry, only names of geographic units affected by disaster. In addition, a connection key is available for connecting the dataset to the PRIO-GRID data frame^20^. PRIO-GRID is a unified global grid structure aiding the compilation, management, and analysis of spatial data, and consists of quadratic grid cells that jointly cover all terrestrial areas of the world. Table 1 provides an overview of the indicators included in the GDIS dataset.

**Table 1:** Variable overview and description.

Variable Name	Description
disasterno	ID-variable from EM-DAT^6^, use this to join the geocoded data with EM-DAT records to obtain information on the specific disasters
id	ID-variable identifying each disaster in the geocoded dataset. Contrary to *disasterno* each disaster in each country has a unique id number
geo_id	Unique ID-variable for each location
country	Name of the country within which the location is
iso3	Three-letter country code, ISO 3166–1
gwno	Gledistsch and Ward country code^32^
geolocation	Name of the location of the observation, which corresponds to the highest (most disaggregated) level available. For instance, observations at the third administrative level will have geolocation values identical to the *adm3* variable
level	The administrative level of the observation, ranges from 1–3 where 3 is the most disaggregated
adm1	Name of administrative level 1 for the given location
adm2	Name of administrative level 2 for the given location
adm3	Name of administrative level 3 for the given location
location	The location as it was extracted from the orginal dataset. This is the string on which the geocoding was based
historical	Marks whether the disaster happened in a country that has since changed, takes the value 1 if the disaster happened in a country that has since changed, and 0 if not
hist_country	Name of country at the time of the disaster, if the observation takes the value 1 on the *historical* variable, this is different from the *country* variable
disastertype	Type of disaster as defined by EM-DAT: flood, storm, earthquake, extreme temperature, landslide, volcanic activity, drought or mass movement (dry)
geometry	Geometry for the observation’s most disaggregated known administrative level. The polygon is extracted from the GADM database^18^ (this variable is not included in the csv-file)
centroid	Centroid of the administrative level, longitude-latitude coordinates

GDIS_disasterlocations.rdata

GDIS_disasterlocations.gpkg

GDIS_disasterlocations.gdb

GDIS_disasterlocations.csv

GDIS-PRIOGRID_key.csv

## Technical Validation

**Fig. 3:**
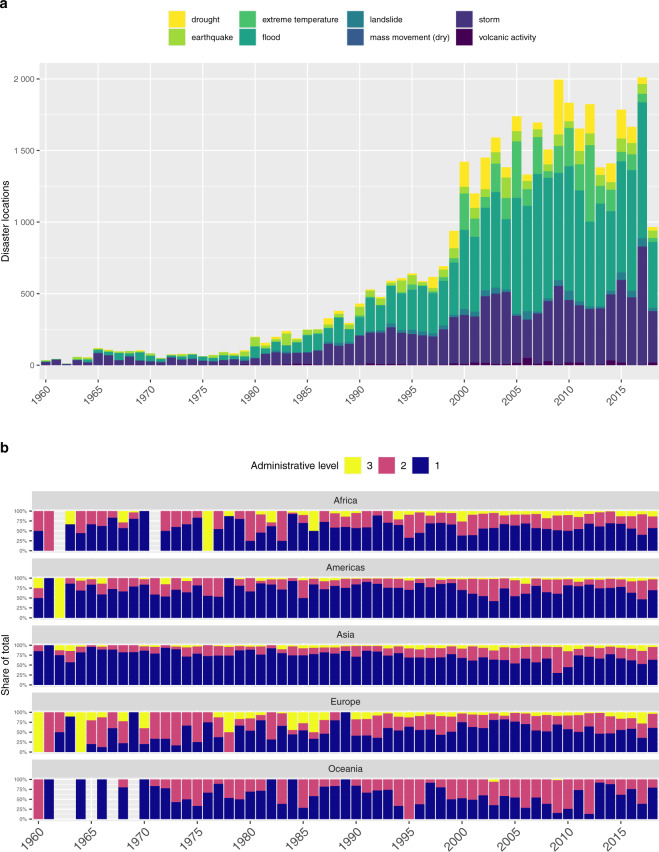
Temporal trends in disaster locations by type (**a**) and continent (**b**). These graphs were created by combining spatial information in GDIS with matching data on disaster type and time from EM-DAT.

A central challenge when working with geographical data relating to historical events is temporal bias in coding. Information about natural hazards and resulting disasters is much more readily available today than in the 1960s, and this is especially true regarding details on the location of remote events in developing countries. Partly for this reason, the EM-DAT disasters exhibit a powerful upward trajectory (Fig. 3a). However, the increasing frequency of disaster events is also due to two other contemporaneous trends: global warming and associated physical processes that have increased the prevalence and severity of natural hazards^21,22^, and population growth and shifting settlement patterns that have led to increasing human exposure to several types of hazards^23–26^. Since the early 2000s, the number of disasters recorded in EM-DAT appears to have stabilized at around 300–350 events per year. A similar stagnation is evident for the human cost of hazards, perhaps reflecting global improvements in coping capacity. However, material losses continue to rise as a result of increasing exposure of economic assets^1,27^.

In order to assess the magnitude of this potential bias for the geocoding-project, we look at the degree to which the share of disaster locations that are ascribed to the second- and third-level administrative unit has increased over time. The vast majority of the geocoded disaster locations are on the first-level administrative unit. The precision of the subnational location information provided in EM-DAT is sensitive to reporting quality and varies across disaster types, countries, and world regions, as well as over time. This fact is well known to CRED, who frequently discuss this and other (data) challenges related to the quality of reporting^28–30^. The uneven coverage of disaster reporting is also investigated by Lorini *et al*.^31^, who – looking at flood coverage in Wikipedia – find a clear skew towards high-income, English-speaking countries. Reassuringly, Fig. 3b shows that the expected rise in locational detail over time is modest. As could be expected, the share of locations at the second and third level appears higher across Europe than the other continents. However, both Africa and Oceania show a relatively stable shares of observations on level 2 and 3 over time. Looking at the totals across all continents, 19% of the disaster locations in GDIS in the 1960s were assigned to second-order units or higher. The corresponding ratio for the years since 2010 is 30%. For most practical purposes, we believe this trend is unlikely to constitute a major problem, but even so, care should be exercised whenever using GDIS data to conduct cross-continent comparisons.

## Usage Notes

The GDIS dataset contains geospatial information on the location of disasters registered in EM-DAT. To obtain additional information on these disasters, such as date, type, and magnitude, the user must consult the EM-DAT database. GDIS can be joined with EM-DAT via the *disasterno* identifier, which is common to both datasets, and this procedure is described in the accompanying R script. The disaster location data in GDIS reflects the information available in the most up-to-date version of EM-DAT at the time of download. The first batch of data was downloaded from EM-DAT in early 2016, with subsequent downloads of additional disasters in 2018 and 2019. As EM-DAT is continuously updated also with information on past events, the tables of disasters in the two datasets may not match perfectly for all countries for all years.

Note also that in order to avoid inflating the number of locations (and hence the size of the data files), only the observation at the highest level of precision is kept in cases where the locations given for one specific disaster are overlapping. This means that the user has to rescale the unit of analysis for observations that are on admin levels 3 and 2 in the event that the desired unit of observation is at a lower (i.e. more aggregate) level. To retrieve geographic information for a less disaggregated administrative level than a given observation provides, the user should consult the GADM boundary data and match the disaster locations with the corresponding geographic polygons available from this database.

## Data Availability

An R script used for the descriptive analyses presented can be found in the SEDAC repository together with the data files. All data presented in tables and figures in the manuscript can be reproduced using the provided code. Note that for Fig. 3 this also requires downloading and joining data from EM-DAT.
